# Supplementary material for: Identification and Phenotype of MAIT Cells in Cattle and Their Response to Bacterial Infections
Source: Front Immunol. 2021 Mar 11;12:627173. doi: 10.3389/fimmu.2021.627173 (PMC7991102; doi:10.3389/fimmu.2021.627173)
Supplement: Supplementary file 2 [file Data_Sheet_2.pdf]

Supplementary table 1. Number of paired sequence reads after quality control and TCR database alignment for TCR alpha (TRA) and beta chain (TRB) sequencing of MAIT ($CD8^+$ MR1-5-OP-RU $tetramer^+$) and non-MAIT ($CD8^+$ MR1-5-OP-RU $tetramer^-$) T cells sorted from PBMC (n = 4).

	**TRA**		**TRB**	
	non-MAIT	MAIT	non-MAIT	MAIT
**Cow 1**	97999	68947	80861	165486
**Cow 2**	95250	34336	33350	43914
**Cow 3**	46090	31828	48989	126600
**Cow 4**	68335	46797	82317	89044

Supplementary table 2. Number of animals per tissue and staining panel for phenotypic characterisation of MAIT cells ($CD8^+$ MR1-5-OP-RU $tetramer^+$) in tissues. Ln Mes. – Mesenteric lymph node. Ln Prescap. – Prescapular lymph node.

	**PBMC**	**Lung**	**Spleen**	**Liver**	**Ileum**	**BAL**	**Ln Mes.**	**Ln Prescap.**
$CD3^+Tet^+$	7	7	7	7	4	7	4	7
$CD8^+Tet^+$	7	7	7	6	4	6	4	7
% $CD45RO^+CCR7^-$	7	5	7	5	4	6	4	7
% CD25 high	7	5	7	5	4	6	4	7
TCR & Co-receptor	7	7	7	6	4	6	4	7
