# Supplementary figures and images for: Identification and Phenotype of MAIT Cells in Cattle and Their Response to Bacterial Infections
Source: Front Immunol. 2021 Mar 11;12:627173. doi: 10.3389/fimmu.2021.627173 (PMC7991102; doi:10.3389/fimmu.2021.627173)

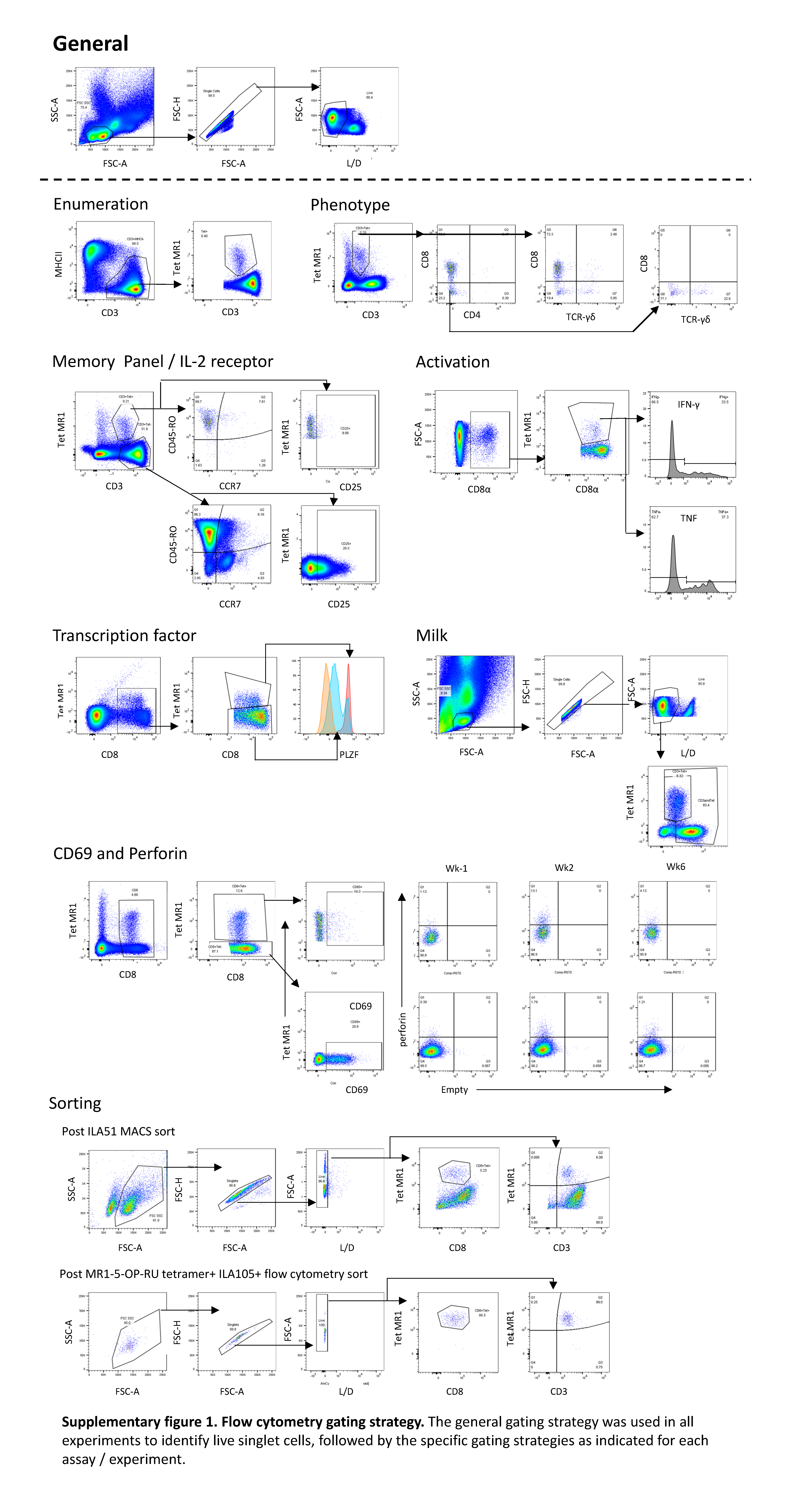

Supplement: Supplementary file 3 [file Image_1.TIF]

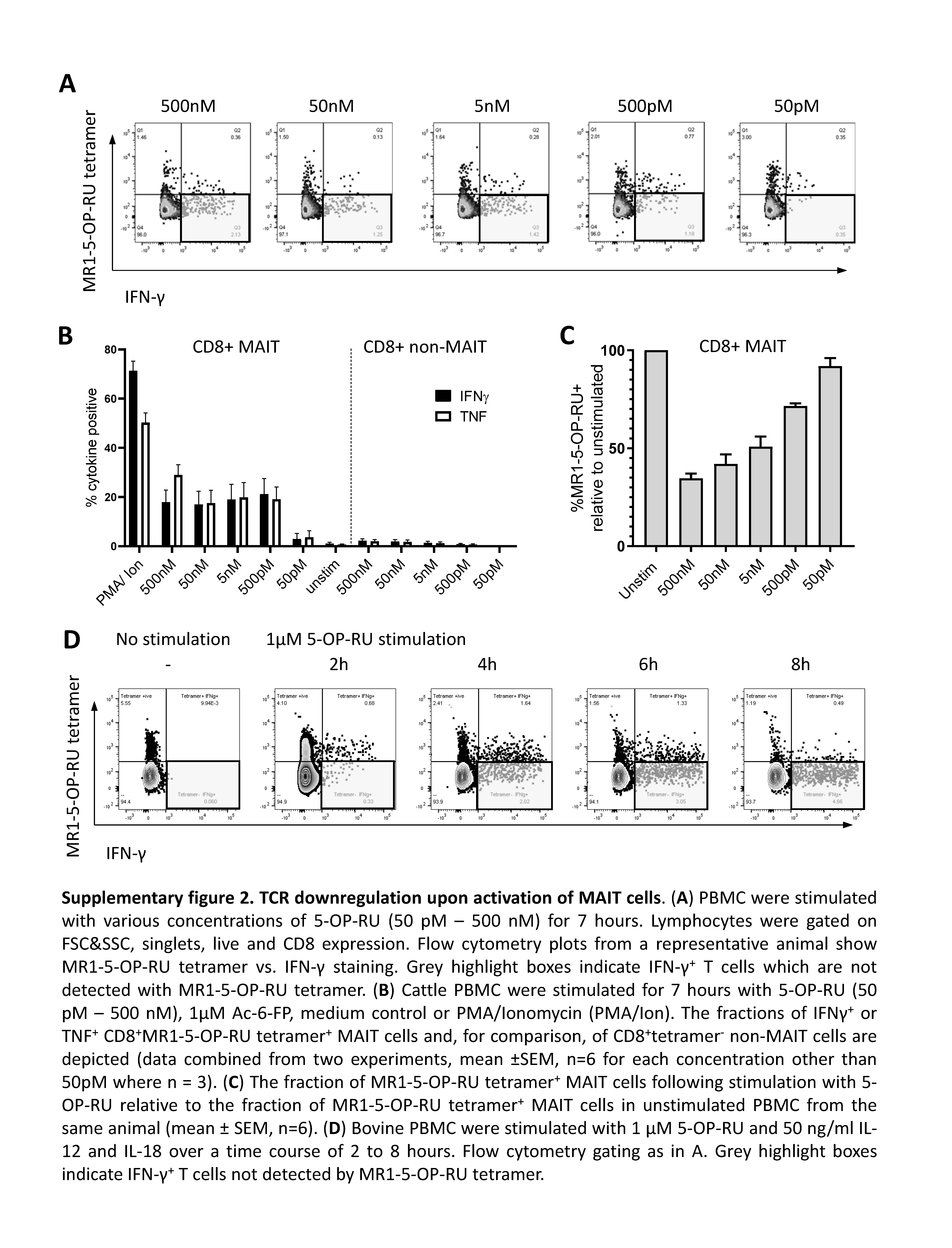

Supplement: Supplementary file 4 [file Image_2.TIF]

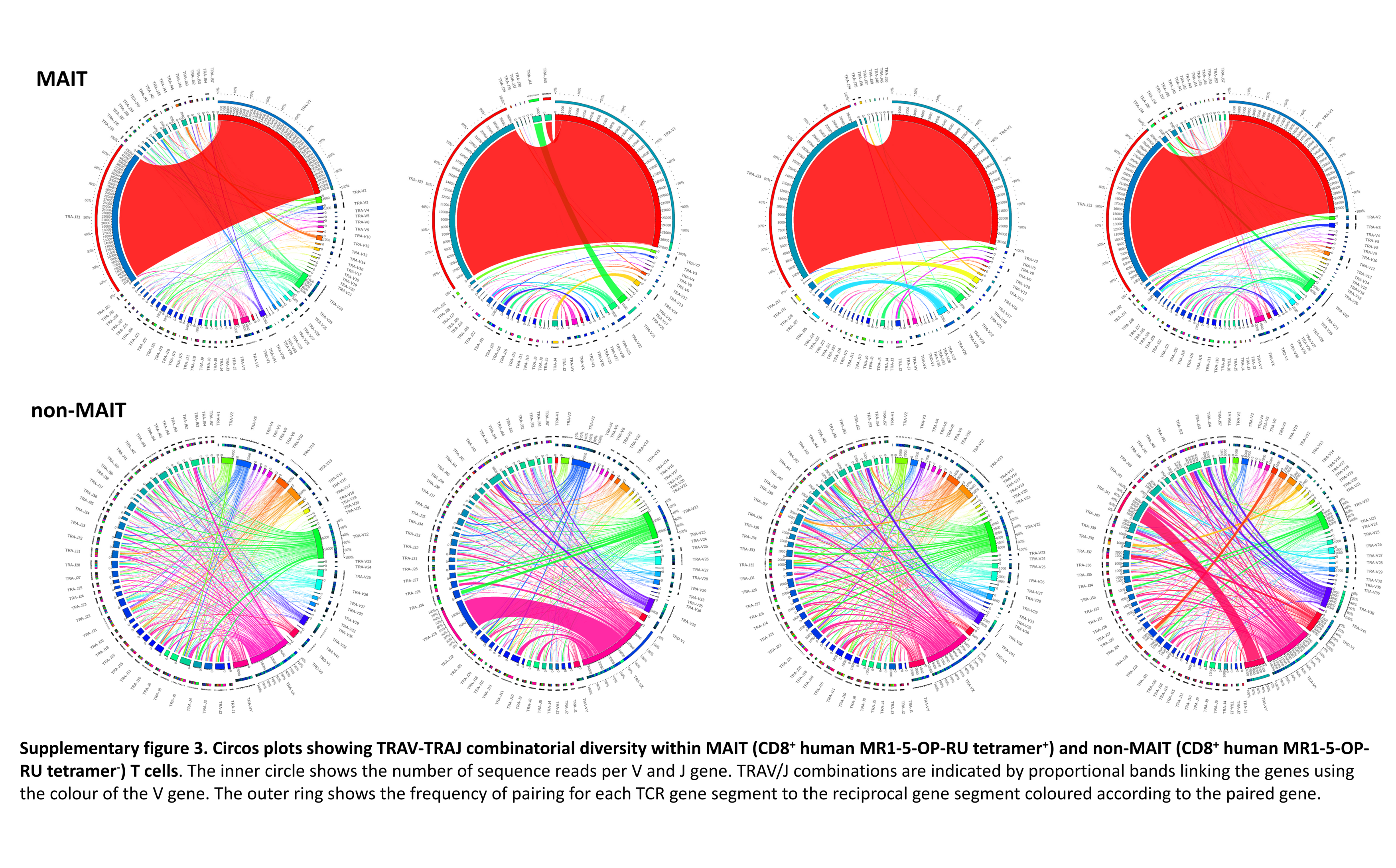

Supplement: Supplementary file 5 [file Image_3.TIF]

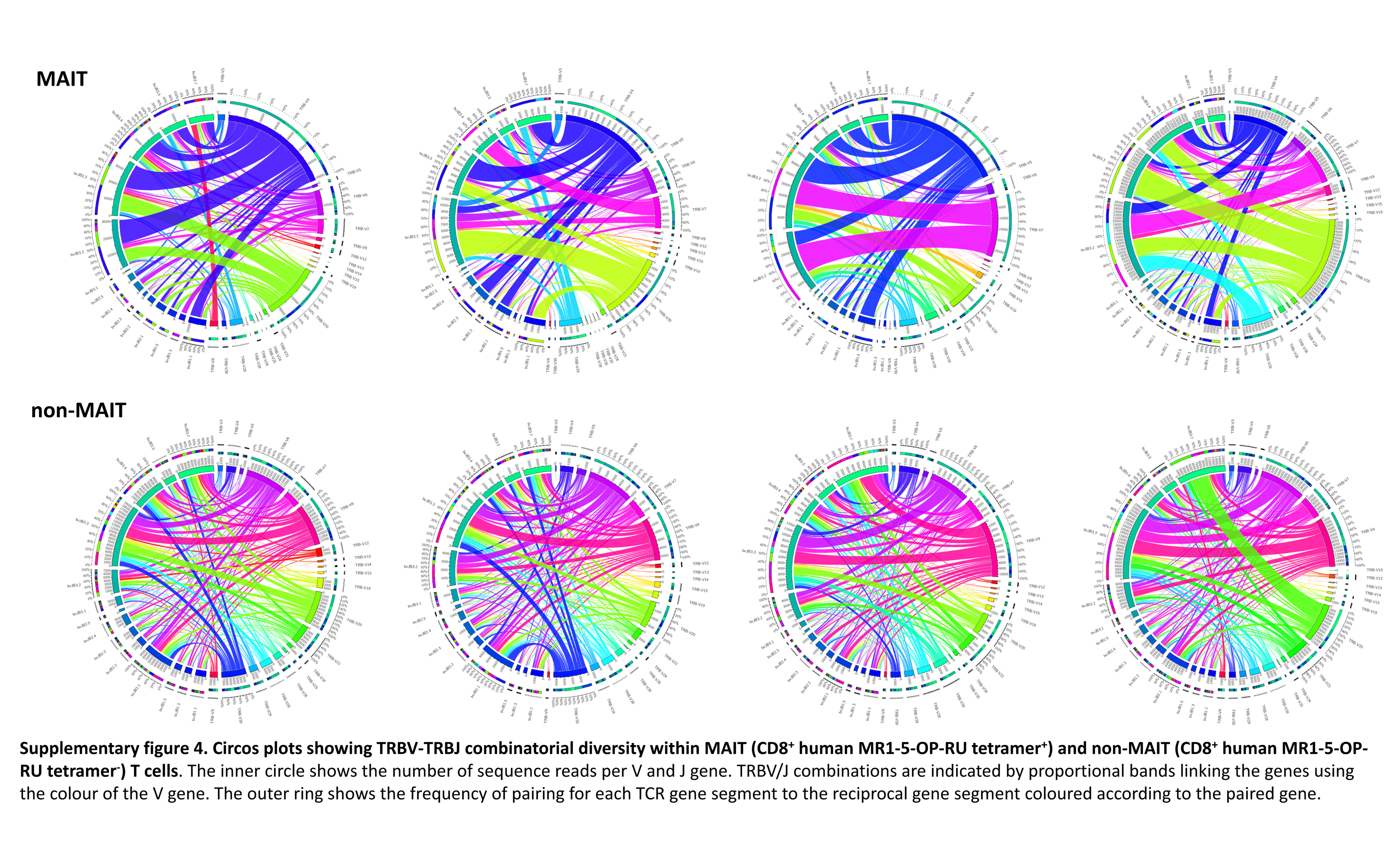

Supplement: Supplementary file 6 [file Image_4.TIF]

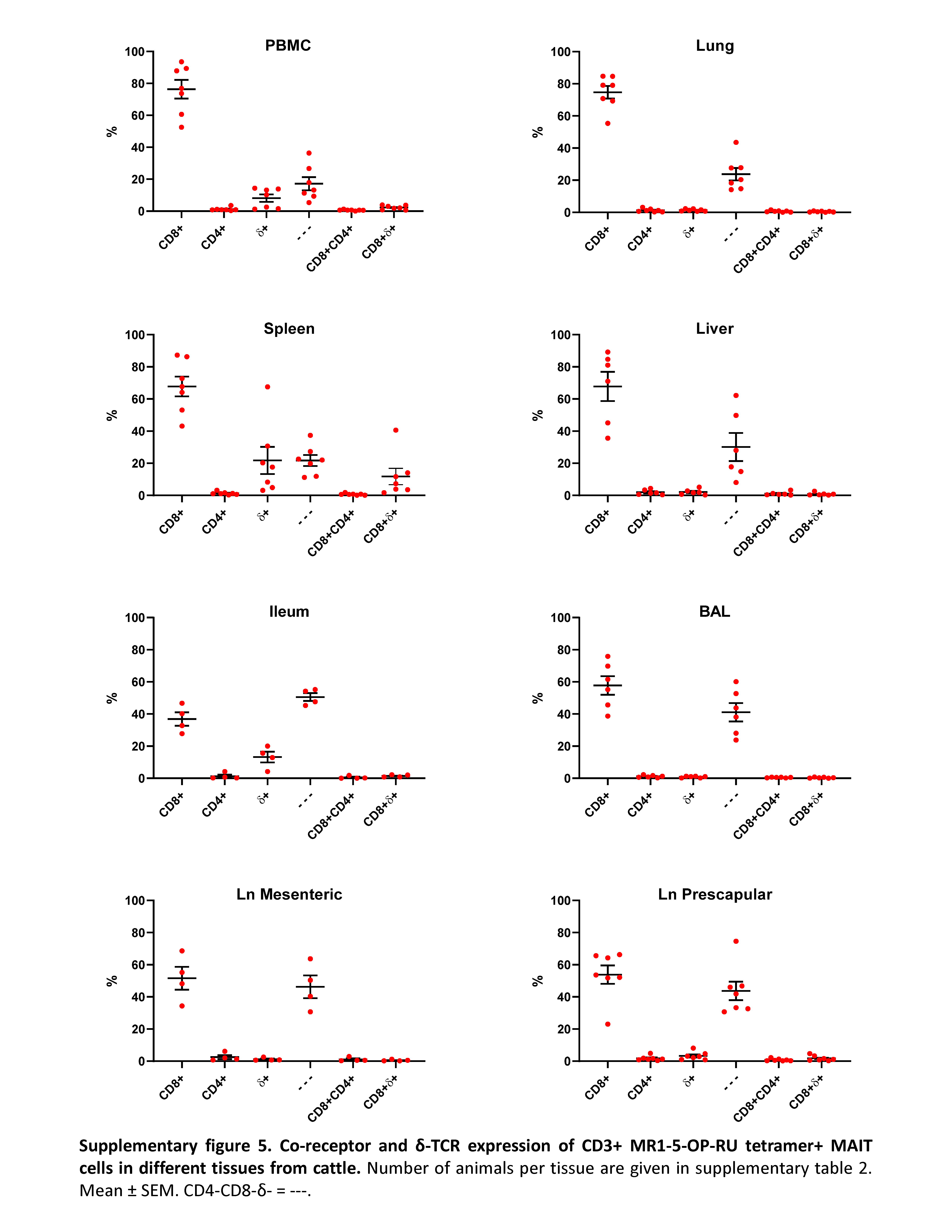

Supplement: Supplementary file 7 [file Image_5.TIFF]

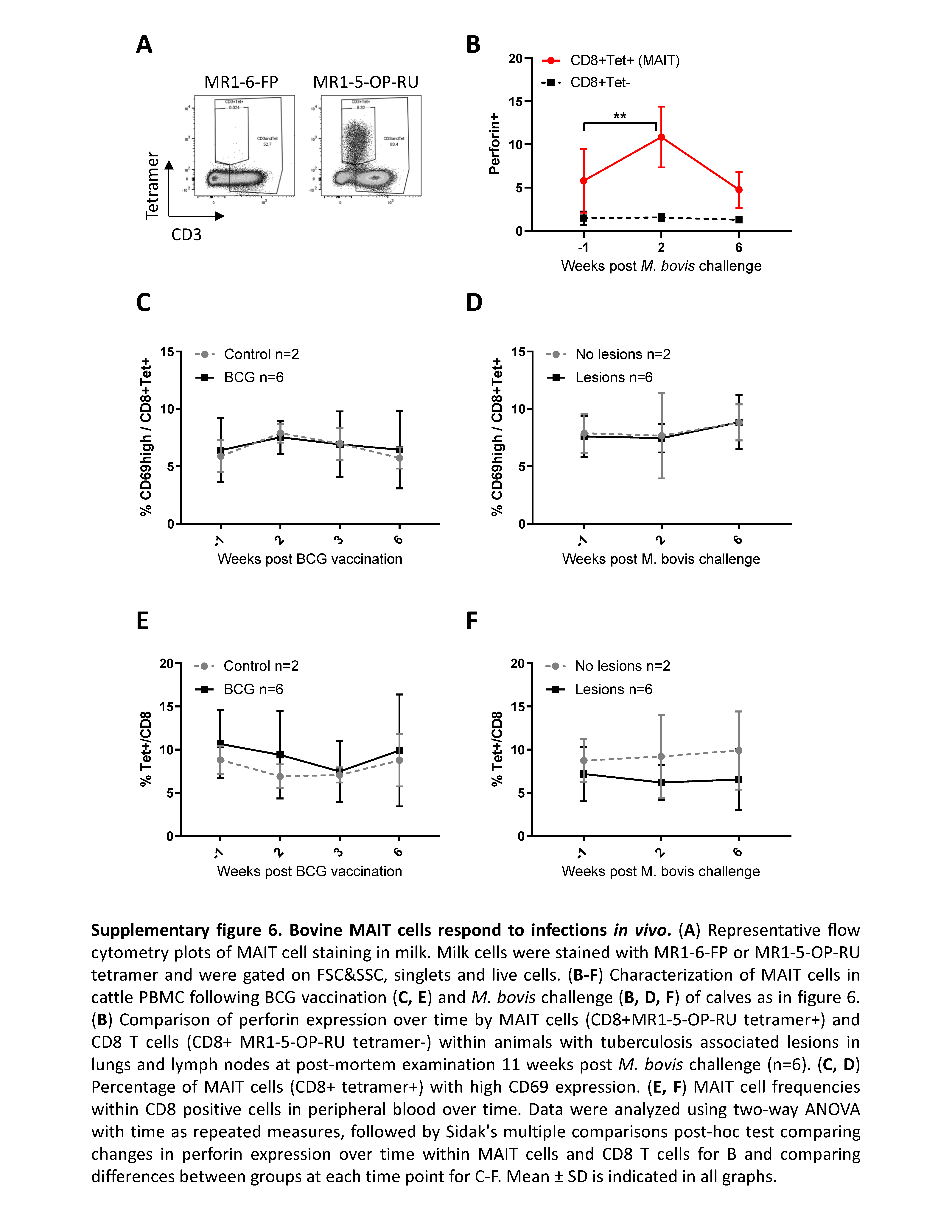

Supplement: Supplementary file 8 [file Image_6.TIFF]

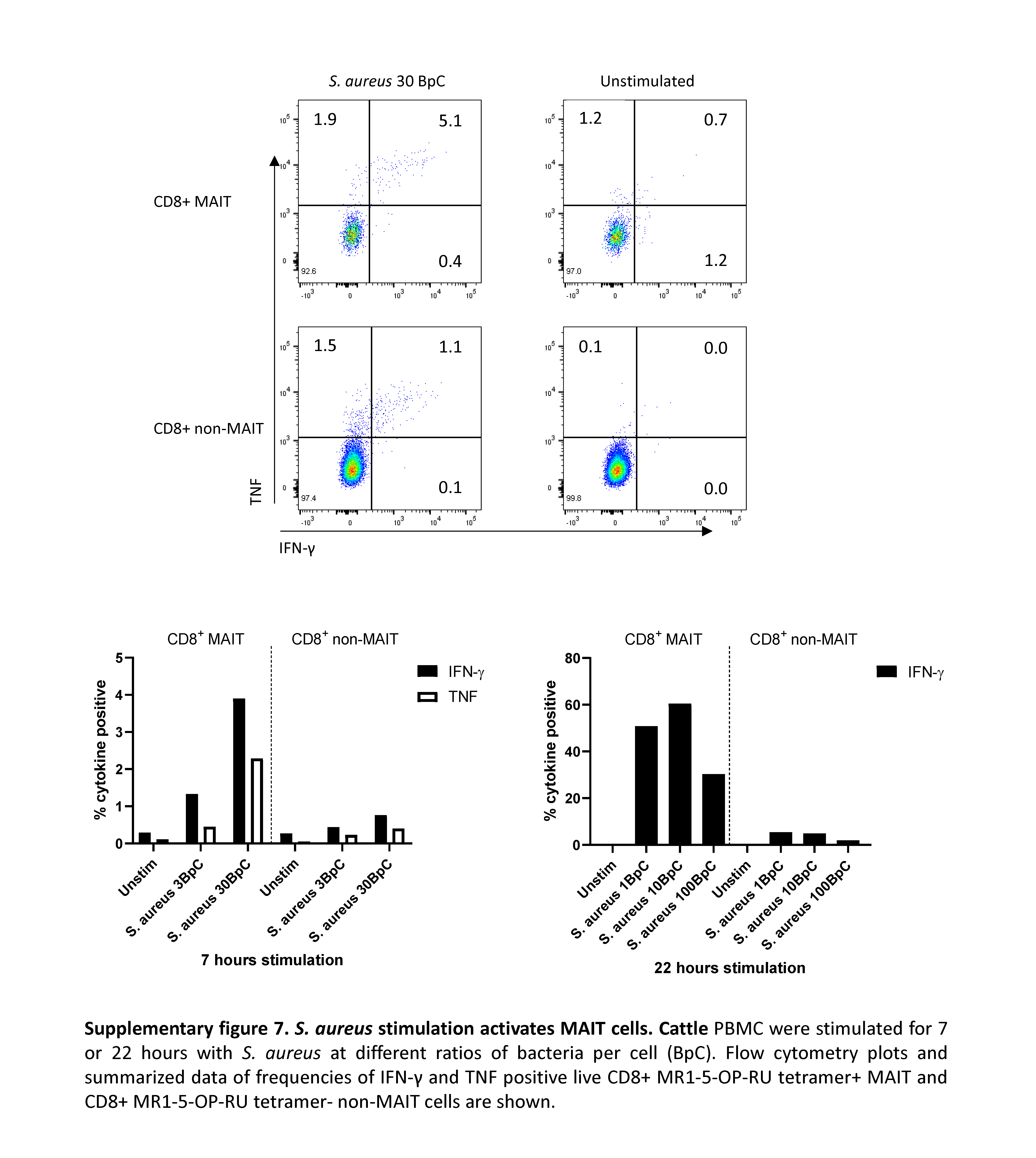

Supplement: Supplementary file 9 [file Image_7.TIFF]
